# The Secreted Lipoprotein, MPT83, of *Mycobacterium tuberculosis* Is Recognized during Human Tuberculosis and Stimulates Protective Immunity in Mice

**DOI:** 10.1371/journal.pone.0034991

**Published:** 2012-05-02

**Authors:** Fan F. Kao, Sultana Mahmuda, Rachel Pinto, James A. Triccas, Nicholas P. West, Warwick J. Britton

**Affiliations:** 1 Mycobacterial Research Program, Centenary Institute, Sydney, New South Wales, Australia; 2 Discipline of Infectious Diseases and Immunology, Central Clinical School, Sydney Medical School, University of Sydney, Sydney, New South Wales, Australia; 3 Discipline of Medicine, Central Clinical School, Sydney Medical School, University of Sydney, Sydney, New South Wales, Australia; University of Cape Town, South Africa

## Abstract

The long-term control of tuberculosis (TB) will require the development of more effective anti-TB vaccines, as the only licensed vaccine, *Mycobacterium bovis* bacille Calmette-Guérin (BCG), has limited protective efficacy against infectious pulmonary TB. Subunit vaccines have an improved safety profile over live, attenuated vaccines, such as BCG, and may be used in immuno-compromised individuals. MPT83 (Rv2873) is a secreted mycobacterial lipoprotein expressed on the surface of *Mycobacterium tuberculosis*. In this study, we examined whether recombinant MPT83 is recognized during human and murine *M. tuberculosis* infection. We assessed the immunogenicity and protective efficacy of MPT83 as a protein vaccine, with monophosphyl lipid A (MPLA) in dimethyl-dioctadecyl ammonium bromide (DDA) as adjuvant, or as a DNA vaccine in C57BL/6 mice and mapped the T cell epitopes with peptide scanning. We demonstrated that rMPT83 was recognised by strong proliferative and Interferon (IFN)-γ-secreting T cell responses in peripheral blood mononuclear cells (PBMC) from patients with active TB, but not from healthy, tuberculin skin test-negative control subjects. MPT83 also stimulated strong IFN-γ T cell responses during experimental murine *M. tuberculosis* infection. Immunization with either rMPT83 in MPLA/DDA or DNA-MPT83 stimulated antigen-specific T cell responses, and we identified MPT83_127–135_ (PTNAAFDKL) as the dominant *H-2^b^*-restricted CD8^+^ T cell epitope within MPT83. Further, immunization of C57BL/6 mice with rMPT83/MPLA/DDA or DNA-MPT83 stimulated significant levels of protection in the lungs and spleens against aerosol challenge with *M. tuberculosis*. Interestingly, immunization with rMPT83 in MPLA/DDA primed for stronger IFN-γ T cell responses to the whole protein following challenge, while DNA-MPT83 primed for stronger CD8^+^ T cell responses to MPT83_127–135_. Therefore MPT83 is a protective T cell antigen commonly recognized during human *M. tuberculosis* infection and should be considered for inclusion in future TB subunit vaccines.

## Introduction

One-third of the world’s population is infected with *Mycobacterium tuberculosis*, the pathogen responsible for tuberculosis (TB). In 2010, the prevalence of active TB cases was estimated to be 178/100,000, resulting in nearly nine millions new cases and 1.5 million deaths worldwide [1]. The incidence has declined steadily at only at a rate 1.3% per year since 2002, despite the implementation of the Directly Observed Therapy – Shortcourse (DOTS) strategy using observed anti-microbial therapy and the extended program of immunization with the only currently-licensed TB vaccine, *Mycobacterium bovis,* bacille Calmette-Guérin (BCG) in high burden countries. Although BCG is effective against severe forms of childhood TB, its protective efficacy against pulmonary TB in adolescents and adults has ranged from 0 to 80% [2]. Several approaches have been taken to either improve the protection afforded by BCG or replace it with a more effective vaccine. Modified BCG vaccines which have entered clinical trials include recombinant BCG expressing listeriolysin (rBCGΔ*ure*::*hly*) [3] and BCG over-expressing Antigen 85B (rBCG30) [4]. Subunit vaccines have the advantage of safety when used in immune-compromised individuals, such as those infected with the human immunodeficiency virus (HIV), and can be used alone or to boost immunity in individuals previously immunized with BCG [5]. Recently a number of subunit vaccines for TB have entered clinical trials, these include fusion proteins based on Antigen (Ag) 85B and ESAT-6 (HyVac1) [6], Ag85B and TB10.4 (HyVac4) [7] and Rv1196 and Rv0125 (Mtb72f) [8]; as well as the non-replication Modified Vaccinia Ankara expressing Ag85A (MVA85A) [9].

These first generation subunit vaccines contain only a small number of the possible *M. tuberculosis* antigens recognised by genetically diverse human populations, and there is the need to expand the repertoire of candidate antigens. Criteria for the inclusion of *M. tuberculosis* proteins in subunit vaccines include their recognition by T cells of *M. tuberculosis*-infected humans, their ability to induce protective immunity in experimental infection, and their suitability for mass production and formulation with adjuvants [10].

MPT83 (Rv2873) is a cell wall-associated lipo-glycoprotein of *M. tuberculosis*, whose function is unknown, although it has been suggested to play a role in adhesion and dissemination based on sequence analysis [11]. Its homologues in *M. bovis*, MPB83 and MBP70, are sero-dominant antigens during *M. bovis* infection in both cattle and badgers [12], [13]. DNA vaccination studies in cattle revealed that MPB83 is immunogenic, generating strong T cell and B cell responses [14]–[16]. Interestingly, pre-treatment of animals with anti-MPB83 antibodies resulted in increased survival during *M. bovis* infection [17]. The expression of MBP83 varies between different strains of BCG owing to a point mutation in the positive regulator, Sigma factor K (SigK) [18]. During *in vitro* culture *M. tuberculosis* expresses relatively small quantities of MPT83 and MPT70, but the expression level of MPT83 is increased during infection [11].

In this study we have investigated the immuno-reactivity of MPT83 in human and experimental tuberculosis, and its capacity to induce protective immunity. The recombinant MPT83 expressed in *Escherichia coli* was recognized by the majority of human TB patients, as well as during murine *M. tuberculosis* infection. DNA vaccine expressing MPT83 elicited antigen-specific IFN-γ secreting T cell responses, and using overlapping peptides spanning the entire sequence of MPT83, the *H-2^b^*-restricted CD4^+^ and CD8^+^ T cell epitopes were identified. Both DNA and protein vaccine formulations generated protective immunity against murine *M. tuberculosis* infection. Therefore MPT83 is an important antigen with defined T cell responses in humans and mice, and should be considered for inclusion in future subunit vaccine candidates.

## Results

### Expression and Characterisation of Recombinant MPT83

**Figure 1:**
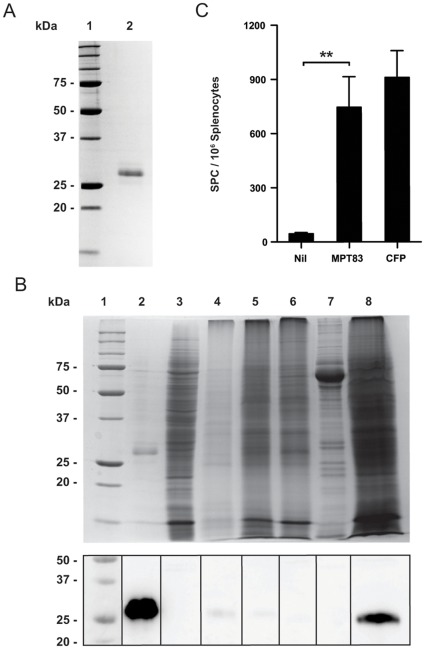
The expression and detection of the recombinant MPT83 protein. Full length *mpt83* was cloned into pET19b and expressed in *E. coli* BL21. (A) Following Co^2+^ affinity chromatography the 28 kDa purified protein was visualized by SDS-PAGE. Lane 1, Molecular weight marker; lane 2, purified rMPT83 500 ng. (B) Coomassie stained SDS-PAGE and anti-MPT83 immunoblot of purified rMPT83 and *M. tuberculosis* sub-cellular fractions. Lane 1, Molecular weight marker; lane 2, purified rMPT83 protein; lane 3, cytoplasmic proteins; lane 4, Tx114 fraction; lane 5, cell wall fraction; lane 6, soluble cell wall fraction; lane 7, culture filtrate proteins; lane 8, whole cell lysate. (C) The number of antigen-specific IFN-γ- secreting T cells (mean±SEM) in splenocytes from *M. tuberculosis*-infected C57BL/6 mice (n = 3) following stimulation with rMPT83, *M. tuberculosis* culture filtrate protein (CFP) and media alone for 16 h. The data are representative of two independent experiments and the statistical significance was determined by the Student’s *t*-test (**, *p*<0.01).

The full length MPT83 was expressed as a recombinant protein in *E. coli* BL21 (DE3), solubilized and purified using the Talon Co^2+^ based metal affinity resin. The purified rMPT83 protein with an N-terminal HIS-tag was refolded, and a single protein with a relative molecular mass (M_r_) of 28 kDa was identified following separation by SDS-PAGE (Fig. 1A). This compares to the predicted molecular mass of 26 kDa for MPT83.

To determine the location of MPT83 in *M. tuberculosis*, high-titre antibodies against MPT83 were raised in mice immunized subcutaneously with rMPT83 and used to probe sub-cellular preparations of *M. tuberculosis* by immunoblotting (Fig. 1B). MPT83 in *M. tuberculosis* fractions was detected as a band at 25 kDa, while the rMPT83 was detected at 28 kDa. MPT83 was abundant in the whole cell lysate (Fig. 1B, lane 8), and weak bands were detected in Tx-114 membrane protein (lane 4) and cell wall protein fractions (Fig. 1B, lane 5 and 6). By contrast, no MPT83 was detected in the fractions containing cytoplasmic proteins (Fig. 1B, lane 3) and culture filtrate proteins (Fig. 1B, lane 7).

### MPT83 is Recognized as a T Cell Antigen During *M. tuberculosis* Infection

To determine if MPT83 is recognized during *M. tuberculosis* infection, splenocytes from *M. tuberculosis* infected mice were stimulated either with rMPT83, or *M. tuberculosis* CFP as positive control. The number of antigen-specific IFN-γ secreting T cells was enumerated by ELIspot assay. rMPT83 protein stimulated a mean antigen-specific IFN-γ response of 746±169 SPC/10^6^ cells, compared to 911±148 SPC/10^6^ cells for CFP-stimulated splenocytes and 45±7.2 SFC/10^6^ cells in unstimulated splenocytes (Fig. 1C).

### Human T Cell Responses to MPT83

**Figure 2:**
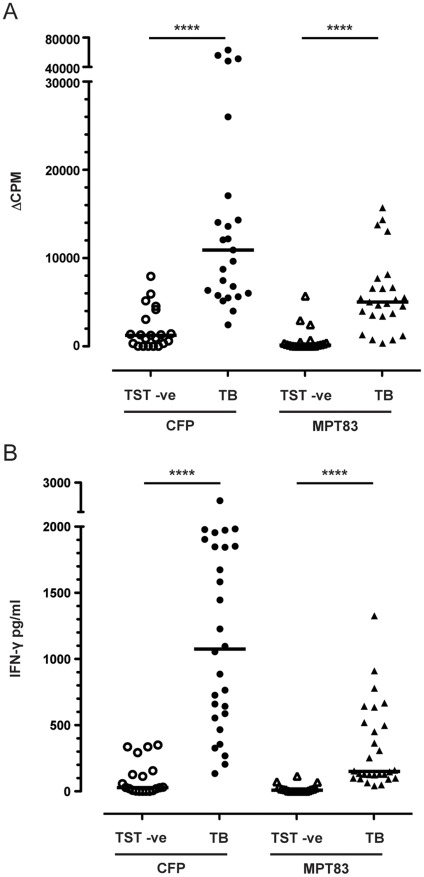
Recognition of MPT83 during *M. tuberculosis* infection in humans. Antigen-specific T cell responses were measured in PBMC from *M. tuberculosis*-infected subjects (TB) (*n* = 28) and tuberculin skin test-negative subjects (TST−ve) (*n* = 20). (A) T cell proliferation in response to *M. tuberculosis* CFP (circles) or rMPT83 (triangles) protein at 10 µg/ml was measured by the net incorporation of ^3^H-thymidine; and (B) T cell production of IFN-γ following antigen stimulation was measured by ELISA. Horizontal lines represent the median for each group. The statistical significances were determined by the Mann-Whitney *U*-test (****, *p*≤0.0001).

*M. bovis* MBP83, which has identical amino acid sequence to MPT83, is highly immunogenic in both cattle and other ruminants. To study whether MPT83 is recognised during human *M. tuberculosis* infection, PBMCs from 28 patients with active TB (13 males, 15 females, ages 22–74) and 20 TST-negative subjects (10 males, 10 females, ages 22–53) were stimulated with rMPT83, and both T cell proliferative and IFN-γ responses were measured. PBMCs from subjects with active TB proliferated significantly more to MPT83 (mean ^3^H-thymidine incorporation 5,869 CPM) than those from TST-negative subjects (641 CPM) (*p<*0.0001), with 20/28 TB patients having an MPT83-specific proliferative response of ΔCPM>3,000 compared to 1/20 TST-negative subjects (Fig. 2A). Similar increases in proliferation were observed when the PBMCs were stimulated with *M. tuberculosis* CFP, with 25/25 TB patients having CFP-specific proliferative response of ΔCPM >3,000 compared to 6/20 TST-negative subjects. Analysis of the culture supernatant revealed significantly higher levels of secreted IFN-γ from the PBMCs of active TB subjects (mean 325 pg/ml) than those of TST-negative subjects (mean 20 pg/ml) following stimulation with rMPT83 (*p<*0.0001) (Fig. 2B). In all, 21/28 TB patients had an MPT83-specific IFN-γ T cell response of >100 pg/ml compared to 1/20 TST-negative subjects. All TB patients had a CFP-specific IFN-γ response of >100 pg/ml compared to 6/20 TST-negative subjects.

### Immunogenicity of DNA-MPT83

**Figure 3:**
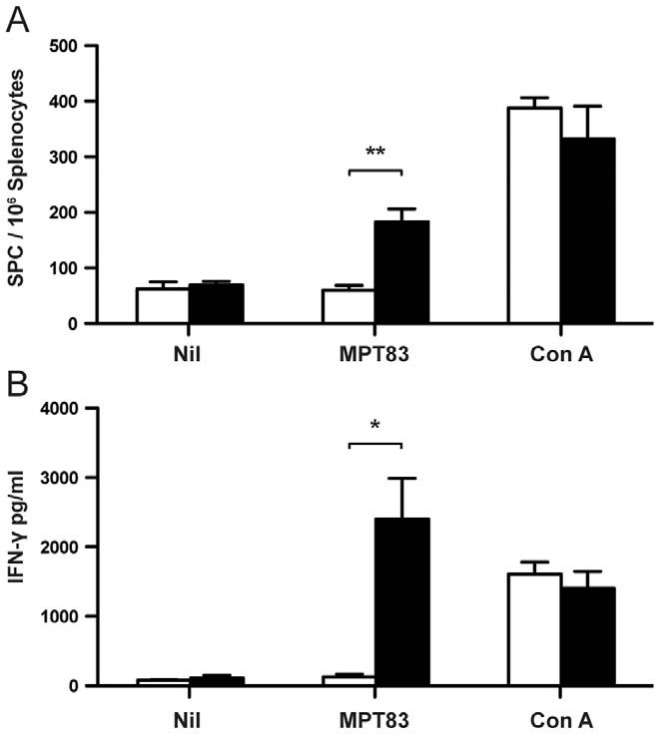
Antigen-specific T cell responses to immunization with DNA-MPT83. C57BL/6 mice (n = 3) were immunized three times at two-weekly intervals with 100 µg DNA-MPT83 or pcDNA3 control vector by i.m.i. Four weeks after the final immunization, splenocytes were harvested and stimulated *ex vivo* with either rMPT83 (10 µg/ml) or Con A (3 µg/ml). (A) The numbers of IFN-γ-secreting cells/10^6^ splenocytes following 16 h of antigen stimulation were enumerated by ELIspot. (B) The levels of IFN-γ released into the supernatants following 72 h of antigen stimulation were measured by IFN-γ ELISA. Data shown are the means±SEM and the statistical significance was determined by the Student’s *t*-test (*, *p*<0.05; **, *p*<0.01), and are representative of three independent experiments.

To investigate the immunogenicity of MPT83, mice were immunized with the DNA vaccine encoding the entire MPT83 protein. Antigen-specific IFN-γ secreting T cells, measured by ELIspot, were significantly more frequent in splenocytes from DNA-MPT83-immunized mice than those from mice immunized with the control vaccine, pcDNA3 (Fig. 3A). Splenocytes were also stimulated with rMPT83 for 72 h, and significantly increased levels of IFN-γ were secreted by splenocytes from DNA-MPT83 immunized mice compared to those from mice immunized with the control DNA vaccine, 2405.90±584.3 pg/ml and 124.45±43.79 pg/ml, respectively (Fig. 3B).

### Murine T Cell EPITOPES within MPT83

**Figure 4:**
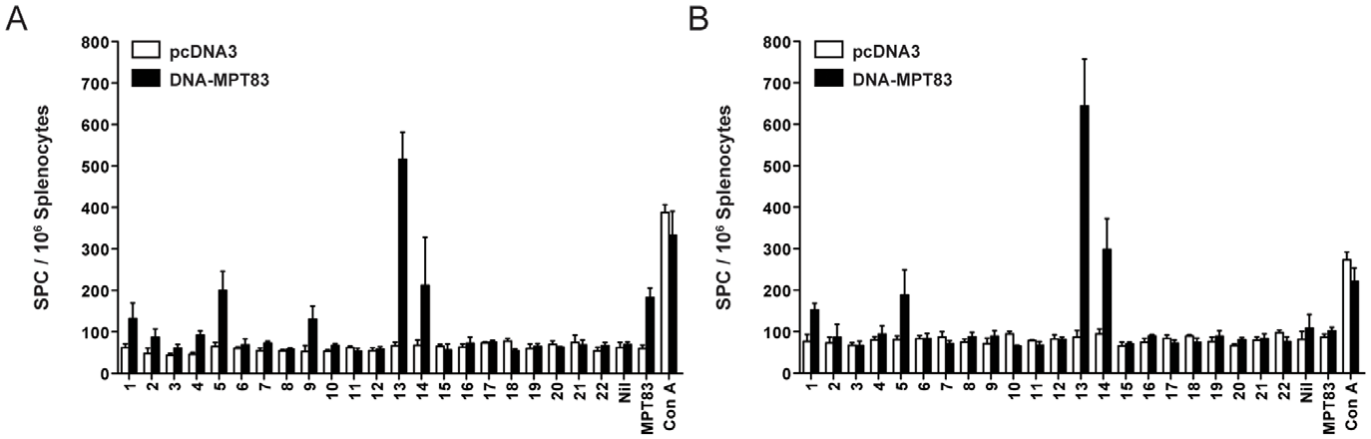
Identification of *H-2^b^*-restricted T cell peptide epitopes in MPT83. C57BL/6 mice (n = 3) were immunized with 100 µg of DNA-MPT83 or pcDNA3 control vector i.m.i three times at two-weekly intervals. Four weeks post-immunization splenocytes were harvested and stimulated *ex vivo* with rMPT83 (10 µg/ml), one of 22 MPT83 15-mer peptides (10 µg/ml) (overlapping by five residues) or Con A (3 µg/ml). (A) The number of peptide-specific and MPT83-specific IFN-γ-secreting T cells were measured by ELIspot after 16 h stimulation; and (B) by ELIspot following depletion of CD4^+^ T cells with the anti-CD4 monoclonal antibody (GK1.5). Data are the means±SEM for replicate samples from the three mice, and are representative of two independent experiments.

Having shown that rMPT83 is recognized by T cells from both *M. tuberculosis*-infected and DNA-MPT83-immunized mice, we next examined the pattern of responses to 22 overlapping 15-mer peptides spanning the length of MPT83 protein in order to map the immunodominant T cell epitopes within MPT83. In C57BL/6 mice immunized with DNA-MPT83, the frequencies of IFN-γ producing cells were the highest in splenocytes stimulated with peptides 13 (MPT83_121–135_) and 14 (MPT83_131–145_) (Fig. 4A). Weaker responses were also observed when cells were stimulated with peptide 9 (MPT83_81–95_), peptide 5 (MPT83_41–55_) and peptide 1 (MPT83_1–15_). When binding of class II MHC molecules to the T cell receptors on CD4^+^ T cells were inhibited using anti-L3T4a mAb (GK1.5), responses to peptides 13 and 14 remained unaffected, while the response to peptide 9 was abolished (Fig. 4B). Weak responses to peptides 1 and 5 were also not affected by CD4^+^ T cell inhibition. This suggests that peptide 9 contains an *H-2^b^*-restricted CD4^+^ T cell epitope and peptides 13 and 14 an *H-2^b^*-restricted CD8^+^ T cell.

**Figure 5:**
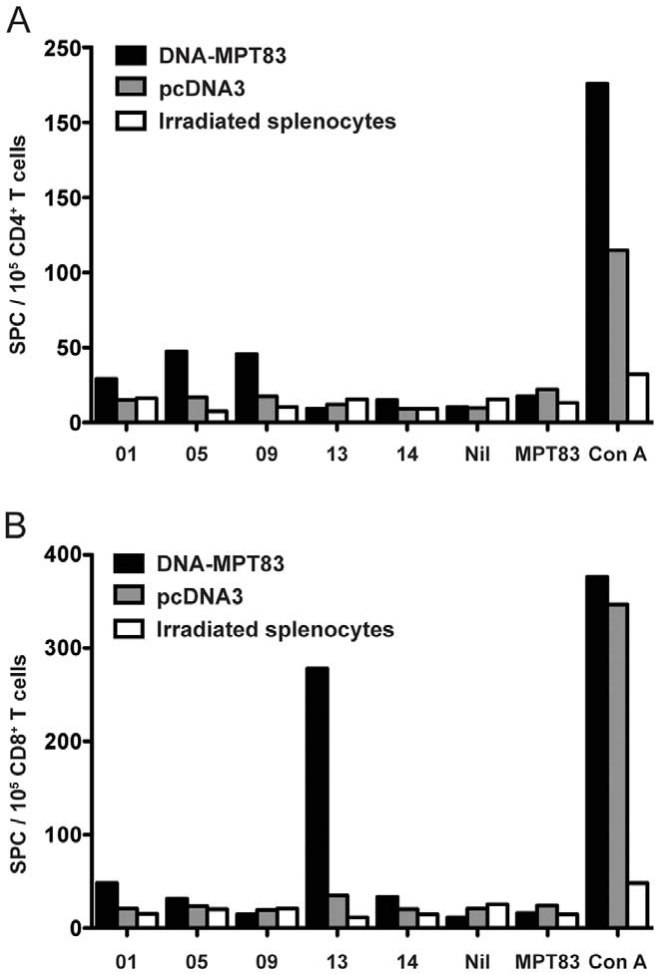
Confirmation of *H-2^b^*-restricted T cell epitopes in MPT83. C57BL/6 mice (n = 3) were immunized with 100 µg of DNA-MPT83 or pcDNA3 control vector by i.m.i three times at two-weekly intervals. Four weeks post-immunization, mice were harvested, and the pooled splenocytes were labelled with anti-CD3-PerCP, anti-CD4-Alexaflour 700 and anti-CD8-APC-Cy7 mAbs and the CD4^+^ and CD8^+^ T cells were separated by flow cytometry. The CD4^+^ (A) and CD8^+^ (B) T cells were stimulated with irradiated splenocytes and the peptides 1, 5, 9, 13 and 14 (10 µg/ml), MPT83 (10 µg/ml) or ConA (3 µg/ml), and the antigen-specific IFN-γ responses were measured by ELIspot after 16 h. Data represent the means of replicates for each treatment, and are representative of two independent experiments.

To confirm the identity of *H-2^b^*-restricted T cell epitopes, CD4^+^ and CD8^+^ T cells from the spleens of DNA-MPT83-immunized mice were purified by flow cytometric sorting and stimulated with irradiated syngeneic splenocytes and peptides 1, 5, 9, 13, and 14, and the IFN-γ T cell responses measured by ELIspot. Modest IFN-γ T cell responses to peptides 5 and 9 were observed with the purified CD4^+^, but not CD8^+^, T cells (Figs. 5A and B). By contrast, a high frequency of IFN-γ producing T cells was observed following stimulation of purified CD8^+^ T cells with peptide 13 alone, and not with the CD4^+^ T cells. Therefore, peptide 13 contains a dominant *H-2^b^*-restricted CD8^+^ T cell epitope, while peptides 5 and 9 may contain CD4^+^ T cell epitopes.

### Minimal *H-2^b^*-restricted CD8^+^ T Cell Epitope in MPT83

**Figure 6:**
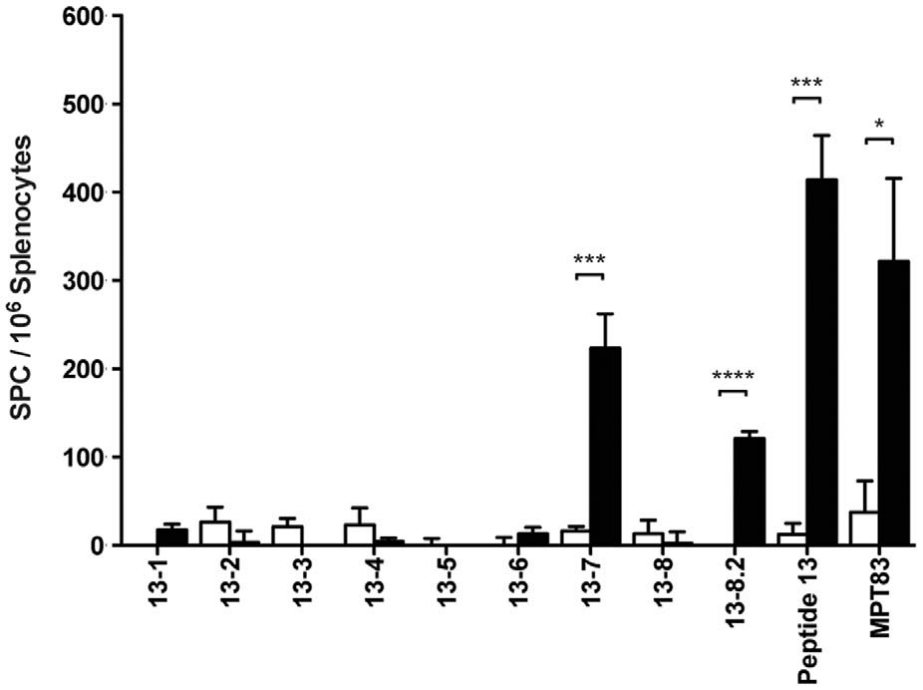
Minimal CD8^+^ T cell epitope within peptide 13 of MPT83. C57BL/6 mice (n = 3) were immunized with 100 µg DNA-MPT83 (▪) or pcDNA3 (□) control vector by i.m.i three times at two-weekly intervals. Four weeks post-immunization, the mice were harvested and the splenocytes were stimulated with nonameric peptides spanning the sequence of peptide 13 and overlapping by one residue (Table 1) at 10 µg/ml, MPT83 (10 µg/ml) and ConA (3 µg/ml) and number of peptide-specific IFN-γ secreting T cells were measured by ELIspot after 16 h. Data shown are the means±SEM for triplicate samples from three mice and representative of two independent experiments. The statistical significance of differences was determined by the Student’s *t*-test (*, *p*<0.05; ***, *p*<0.001, ****, *p*<0.0001).

To resolve further the *H-2^b^*-restricted CD8^+^ T cell epitope, we examined the binding scores of nonameric and octameric peptides within peptide 13 to both *H-2K^b^* and *H-2D^d^* haplotypes of class I MHC molecules using the online prediction algorithm SYFPEITHI. The octameric peptide 13-8.2 (TNAAFDKL) had the highest predicted binding for H-2D^b^ with a score of 22. The nonameric peptides 13-7 (PTNAAFDKL) and 13-5 (FAPTNAAFD) were second and third with binding scores of 12 and 8 respectively. No peptides within peptide 13 were predicted to bind H-2K^b^. Splenocytes from DNA-MPT83 immunized mice were then stimulated with the set of octameric and nonameric peptides within peptide 13, which are summarized in Table 1. Splenocytes from both pcDNA3 and DNA-MPT83 immunized mice responded to stimulation with the mitogen Con A. Only two out of the nine peptides induced IFN-γ T cell responses. Peptide 13-7 stimulation induced the greatest frequency of IFN-γ producing cells, followed by peptide 13-8.2. In both instances the frequencies of IFN-γ producing cells were not as high as those induced by stimulation with the 15-mer peptide 13 (Fig. 6).

**Table 1:** Sequence of overlapping peptides spanning peptide 13_(121–135)_ of MPT83 and the predicted scores* for binding of the peptides to H-2K^b^ and H-2D^b^.

Peptide name	Sequence	SYFPEITHI scores*	
		H-2K^b^	H-2D^b^
13	EYTVFAPTNAAFDKL	-	-
13-1	EYTVFAPTN	0	3
13-2	YTVFAPTNA	0	4
13-3	TVFAPTNAA	0	0
13-4	VFAPTNAAF	0	8
13-5	FAPTNAAFD	0	21
13-6	APTNAAFDK	0	2
13-7	PTNAAFDKL	0	12
13-8	TNAAFDKLA	0	2
13-8.2	TNAAFDKL	-	22

* Predicted binding scores from the website: http://www.syfpeithi.de/Scripts/MHCServer.dll/EpitopePrediction.htm.

### Protective Efficacy of MPT83 DNA and Protein Vaccines

**Figure 7:**
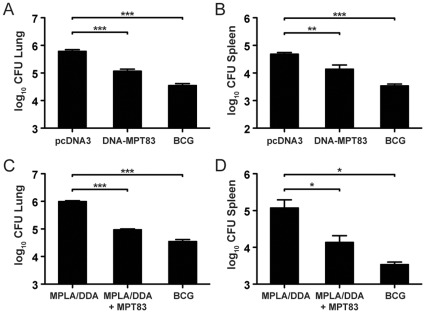
Protective efficacy induced by DNA-MPT83 and rMPT83 immunization. C57BL/6 mice (n = 5) were immunized with 100 µg of DNA-MPT83 and control vector pcDNA3 by i.m.i. (Panels A and B) or 10 µg of rMPT83 in MPLA/DDA by s.c.i. (Panels C and D) three times at two-weekly intervals. Six weeks following the final immunization, mice were infected by aerosol with *M. tuberculosis* H37Rv. Additional mice were immunized with 5×10^5^ CFU of BCG once by s.c.i. 12 weeks before challenge with *M. tuberculosis* H37Rv. After 28 days, mice were sacrificed, and the bacterial loads in the lung and the spleen were determined by CFU enumeration following culture on Middlebrook 7H11 media. The data are the means ± SEM and are representative of three independent experiments for DNA-MPT83 and two experiments for rMPT83. The statistical significance between groups was determined by analysis of variance (*, *p*<0.05, **, *p*<0.01, ***, *p*<0.001).

**Figure 8:**
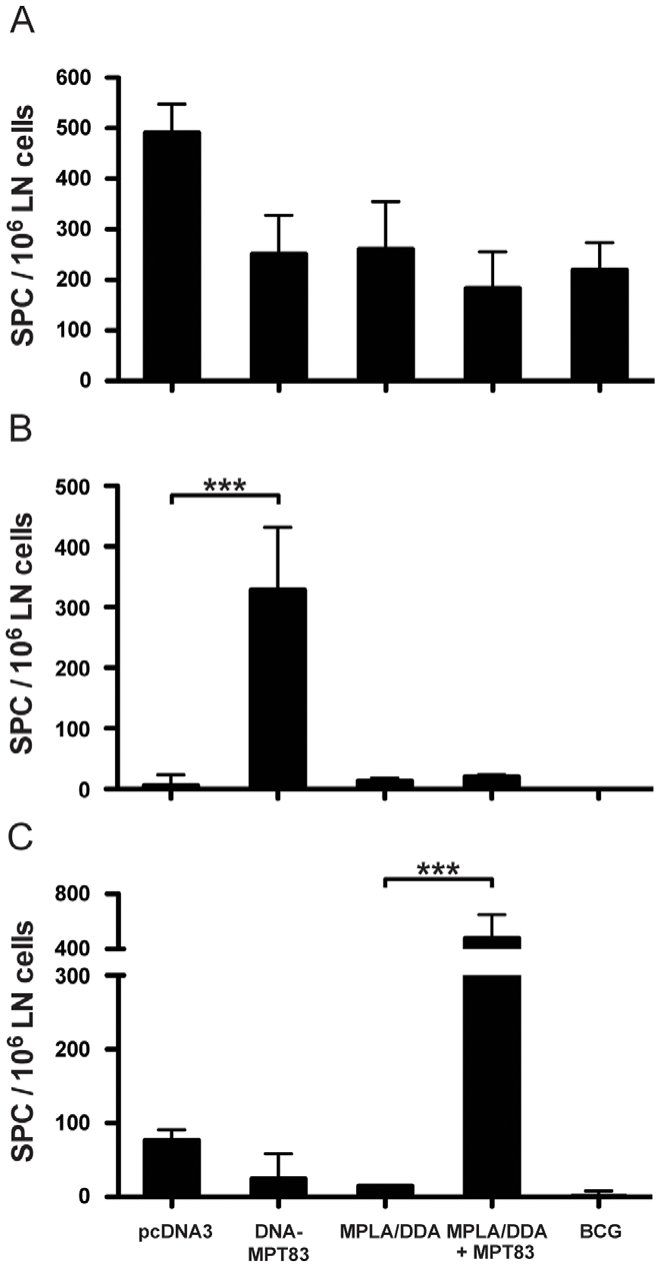
T cell responses following immunization with DNA-MPT83 and rMPT83, and *M. tuberculosis* challenge. C57BL/6 mice (n = 5) were immunized with 100 µg of DNA-MPT83 and control vector pcDNA3 by i.m.i. or 10 µg of rMPT83 in MPLA/DDA by s.c.i. three times at two-weekly intervals. Six weeks following the final immunization, mice were infected by aerosol with *M. tuberculosis* H37Rv. Additional mice were immunized with 5×10^5^ CFU of BCG once by s.c.i. 12 weeks before challenge with *M. tuberculosis* H37Rv. After 28 days, mice were sacrificed, and the MLN was harvested and stimulated *ex vivo* with CFP at 3 µg/ml (A), Peptide 13 at 10 µg/ml (B), and MPT83 at 10 µg/ml (C). The data are the mean±SEM and are representative of two experiments. The statistical significance between groups was determined by analysis of variance (***, *p*<0.001).

The protective efficacy of MPT83 as DNA and protein vaccine was assessed. Following three immunizations with DNA-MPT83 or control DNA vaccine and six weeks rest, C57BL/6 mice were infected with *M. tuberculosis* by aerosol, and the bacterial loads in the lung and spleen were enumerated four weeks after infection. Immunization with DNA-MPT83 conferred significant protection in both the lungs (*p*<0.001) and the spleen (*p*<0.001) compared to control DNA vaccine (Figs. 7A and B). C57BL/6 mice were also immunized with rMPT83 in MPLA/DDA adjuvant or adjuvant alone three times, rested six weeks and challenged with *M. tuberculosis.* The recombinant protein in MPLA/DDA also stimulated significant protection compared to adjuvant alone, with at least 1.0 log_10_ protection in both the lung (*p*<0.0001) and the spleen (*p*<0.05) (Figs. 7C and D). BCG immunization also induced protection in both the lung and spleen (Fig. 7). Antigen-specific T cell responses in the draining MLN were also assessed after *M. tuberculosis* challenge. Both the immunized and control mice developed IFN-γ responses to *M. tuberculosis* CFP (Fig. 8A). Mice immunized with DNA-MPT83, but not control DNA or MPT83/MPLA/DDA, (Fig. 8B) were primed for a strong T cell response to the defined CD8^+^ T cell epitope, MPT83_127–135_. By contrast, mice immunized with MPT83/MPLA/DDA and infected with *M. tuberculosis* developed a high frequency of antigen-specific IFN-γ T cells to the whole MPT83 protein, consistent with a CD4^+^ T cell response, and no significant response to the defined CD8^+^ T cell epitope (Fig. 8C).

## Discussion

*M. tuberculosis* proteins to be considered for inclusion in anti-TB subunit vaccines must be immunogenic in the majority of TB patients, lack cross-reactivity with human proteins, and stimulate protective immunity in experimental models of *M.* tuberculosis infection. To date, there are six subunit vaccines based on one or two mycobacterial proteins with these characteristics that have entered phase I and II clinical trials [19]–[22]. This study has identified MPT83 as an additional candidate with similar features to be considered for inclusion in anti-TB vaccines. MPT83 was recognised during both murine and human *M. tuberculosis* infection. Peripheral blood T cells from the majority of patients with active TB, but not from tuberculin-negative healthy subjects, proliferated and secreted IFN-γ in response to rMPT83. Furthermore, we have demonstrated the protective efficacy of both plasmid DNA expressing MPT83 and rMPT83 protein vaccine in adjuvant against aerosol infection with *M. tuberculosis* in mice.

Expression of *Rv2873* with an additional N-terminal His-Tag in the pET19b system yielded a recombinant protein with the predicted molecular weight of 25.61 kDa, compared to 22.07 kDa for the amino acid sequence of MPT83. Interestingly, probing different sub-cellular fractions of *M. tuberculosis* with mouse anti-rMPT83 antisera detected a protein band with a relative molecular mass of 25 kDa. This is consistent with previous reports that native MPT83 has a M_r_ of 26 kDa in SDS-PAGE, in part due to glycosylation [23]. In this study MPT83 was detected in the whole cell lysate (Fig. 1B, lane 8) and Triton X-114 detergent soluble fractions of *M. tuberculosis*, suggesting MPT83 is an integral membrane protein (Fig. 1B, lane 4). This mirrors studies of MPB83, which reported its presence in the Triton X-114 soluble fraction [24]. MPB83 has also been detected on the surface of *M. bovis* by flow cytometry using monoclonal antibodies [25].

The *M. bovis* MPB83 and the closely related protein MPB70 are major antigens recognised during *M. bovis* infection of cattle, acquired by both natural and experimental transmission. Ninety percent of *M. bovis* infected cattle develop antibody responses against MPB83 [26], while 78% of develop delayed-type hypersensitivity responses to a protein cocktail containing MPB83 [27]. Furthermore, badgers are a natural reservoir for *M. bovis* infection and MPB83 stimulates a strong antibody response in the majority of infected animals [13], [28]. Both MPB83 and MPB70 are abundantly expressed in *M. bovis*, however MPB83 is retained in the cell membrane, while MPT70 is secreted into the culture filtrate [11]. There is variable expression of MPB83 in different strains of *M. bovis* BCG, caused by point mutations in both *sigK* [18] and Rv0444c [29], encoding the positive regulator and repressor that control the SigK regulon containing both *mpb83* and *mpb70*. The wild-type Rv0444c protein in *M. tuberculosis* is associated with moderate to low levels of expression of MPT83 in bacterial cultures [29]. Nevertheless, MPT83 was readily detected in both the whole cell lysate of *M. tuberculosis* H37Rv and the membrane integral proteins fraction. It is also apparent that the protein is expressed during infection *in vivo*, as both TB patients and *M. tuberculosis*-infected mice developed strong IFN-γ T cell responses against MPT83. While the current study examined the recognition of MPT83 in patients with active TB, a recent study of both BCG-vaccinated and *M. tuberculosis*-infected healthy subjects in Kuwait reported the recognition of multiple MPT83-derived peptides by the majority of subjects, resulting in antigen-induced proliferation and IFN-γ production [30].

Immunization of C57BL/6 mice with MPT83 as either a DNA or protein vaccine stimulated strong antigen-specific IFN-γ secreting T cell responses. Similar levels of immunogenicity have been observed in BALB/c mice immunized with RNA encoding MPT83 [31]. CD4^+^ T cells are essential for protective immunity against *M. tuberculosis* infection, but they are also critical for the activation and licensing of CD8^+^ T cell responses. *M. tuberculosis-*specific CD8^+^ T cells contribute to protection, especially during the chronic phase of experimental infection in mice [32], [33]. Overlapping 15mer peptides spanning the MPT83 sequence were used to locate the dominant H-2^b^-restricted CD8^+^ T cell epitope within the peptide MPT83_121–135_, and the minimal epitope defined as MPT83_127–135_, PTNAAFDKL (peptide 13–7). This peptide had the second highest binding score for nonamers binding to H-2D^b^ in both the SYFPEITHI and IEDB epitope prediction algorithms (Table 1). Immunization with DNA-MPT83 primed for strong CD8^+^ T cell responses to the MPT83_127–135_ peptide following *M. tuberculosis* challenge (Fig. 8B). Computational analysis of MPT83 has identified peptides with the potential for binding to multiple human MHC Class I alleles, suggesting human CD8^+^ T cell epitopes my also exist in the protein [30].

Given that MPT83 was recognized during both human and murine *M. tuberculosis* infections, we examined whether the robust T cell responses induced by MPT83 translated into protection against *M. tuberculosis* challenge. Immunization with either rMPT83 in MPLA/DDA adjuvant emulsion or plasmid encoding MPT83 significantly reduced both pulmonary and splenic bacterial loads following *M. tuberculosis* challenge. This effect is consistent with the protection against *M. tuberculosis* infection induced by an RNA-based vaccine encoding MPT83 in BALB/c mice [31]. Furthermore, combinations of DNA vaccines, which included MPT83 as one of several secreted proteins, were also protective against experimental TB infection [34], [35]. DNA immunization using the *M. bovis* homolog, MPB83 protected BALB/c mice against *M. bovis* challenge as well as stimulating T cell responses in immunized cattle [15]. When tested in the guinea pig model of *M. bovis* infection, plasmid MPB83 significantly reduced the extent of granulomatous infiltration in the lungs, although it did not reduce the bacterial load following aerogenic infection [36]. Early studies with DNA vaccines suggested they may have limited applicability in humans, but more recent investigations administrating DNA vaccines into humans using *in vivo* electroporation resulted in significantly enhanced DNA delivery and stimulated robust cellular immune responses, indicating a potential role of DNA vaccination in humans [37], [38]. This study also demonstrated that immunization with rMPT83 protein in MPLA/DDA liposomes was unequivocally protective. The level of protection was considerable, with approximately one log_10_ reduction in bacterial load induced by rMPT83 (Fig. 7), similar level to the protection afforded by other *M. tuberculosis* protein vaccines, which are currently in phase I clinical trials [21], [39], [40].

In general, the level of protection stimulated by rMPT83 and other *M. tuberculosis* protein vaccines is less than that induced by BCG, although they have the advantage of increased safety over BCG in immune-compromised subjects [41]. The potential application of these subunit vaccines is for boosting the effects of infant BCG immunization, or, as demonstrated with DNA encoding Ag85B, for priming prior to BCG to increase the protection afforded by BCG vaccination [42], [43]. The adjuvant used in the current study, MPLA, is a potent inducer of Th1 responses through the activation of TLR-4 [44]. Interestingly, rMPT83/MPLA/DDA primed for stronger IFN-γ T cell responses to the whole protein following challenge, while DNA-MPT83 primed for stronger CD8^+^ T cell responses to MPT83_127–135_, even though they had similar protective efficacies. The development of more effective and safe adjuvants for protein anti-TB vaccines, such as the mycobacterial cell wall component trehalose-6,6-dimycolate and its synthetic analogue trehalose-6,6-dibehenate, which induce both Th1 and Th17 responses [45]–[47], may further increase the protective efficacy and durability of these vaccines. MPT83 is an additional protective antigen, which should be considered for inclusion in future anti-TB vaccines.

## Materials and Methods

### Ethics Statement

All animal experimental protocols and procedures were approved by the Animal Ethics Committee of the University of Sydney with the reference number K75/5-2008/3/4849. The blood samples were collected with informed written consent from patients and ethical approval for the clinical investigations was obtained from the Sydney South West Area Health Service Human Ethics Committee.

### Mice

Six to eight weeks old female C57BL/6 mice were obtained from the Animal Resource Centre (Perth, Australia) and maintained under specific pathogen-free conditions at the animal facility at the Centenary Institute. All mice were housed in temperature-controlled rooms with unrestricted access to food and water.

### Bacterial Strains and Experimental Infection

*M. tuberculosis* H37Rv (ATCC 27294) and *M. bovis* BCG Pasteur were grown in Middlebrook 7H9 broth media supplemented with 10% Middlebrook ADC enrichment (Difco, Detroit, MI) and 0.05% polysorbate 80 (Sigma, St Louis, MO). Cultures were grown to mid-log phase then stored at −80°C. Frozen aliquots of *M. tuberculosis* were resuspended in distilled water for aerosol infection. Mice were infected with approximately 100 CFU of *M. tuberculosis* using a Middlebrook airborne infection apparatus (Glas-Col, Terre Haute, IN), and the infectious dose was confirmed by plating serial dilutions of the lung homogenates harvested 24 h after infection. Mice were immunized with 5×10^5^ CFU of *M. bovis* BCG in sterile PBS by subcutaneous injection (s.c.i.) at the base of the tail. The bacterial loads in the lung and spleen of all infected mice were determined by plating serial dilutions of the tissue homogenate onto Middlebrook 7H11 Bacto agar, supplemented with 10% OADC (Difco), and incubated at 37°C in a humidified incubator for 21 days.

### Construction of Plasmid Vectors

The MPT83 expression vector was constructed with the complete coding sequence of MPT83 (Rv2873). The full-length open reading frame was amplified from H37Rv genomic DNA using the forward primer 5′-GAATTCATGATCAACGTTCAGGCC-3′ and the reverse primer 5′-GATATCTATCACGTGATCTATCGGC-3′. The amplicon was cloned into an intermediate vector (pCR2.1, Invitrogen). The complete MPT83 gene was excised using *EcoR*I and *EcoR*V and blunt ligated into a *Bam*HI digested expression vector, pET19b (Novagen, Madison, WI) carrying an N-terminal histidine tag, to form pSM2. The MPT83 DNA vaccine was constructed by inserting the whole MPT83 gene into the eukaryotic expression vector pcDNA3 (Invitrogen, Carlsbad, CA) under the control of the CMV IE promoter to yield DNA-MPT83. The pcDNA3 vector served as control vaccine.

### Recombinant Protein Expression and Purification

Recombinant MPT83 expression from pSM2 was driven by the T7 in *E. coli* BL21 (DE3). For large-scale production of recombinant protein, an overnight starter culture was used to seed Luria Bertani (LB) broth (Difco) supplemented with ampicillin (100 µg/ml) and incubated at 37°C for 16 h with agitation. This resulted in the over-expression of cytoplasmic MPT83 protein with an N-terminal His-tag that aggregated as insoluble inclusion bodies. The recombinant protein was purified by solubilizing the inclusion bodies in 100 ml of 8 M urea buffer (8 M urea, 0.3 M NaCl, Sodium Phosphate buffer pH 7.6) per litre of culture and slowly agitating at 4°C. The solubilized protein was purified by a Talon Co^2+^ charged immobilized metal affinity chromatography (IMAC) system (Clontech, Mountain View, CA), according to the manufacturer’s instructions, and dialysed against Tris buffer (50 mM, pH 7.5).

### SDS-PAGE and Immunoblotting

Denatured samples were separated on a 12% polyacrylamide gel and proteins were visualised by Coomassie blue (Sigma) staining. For protein immunoblotting, following electrophoresis, proteins were transferred to a BioTrace PVDF 0.45 µm pore sized membrane (Pall, Pensacola, FL) with a Trans Blot Cell (Biorad). The resulting membrane was blocked with 5% skim milk in PBS with 0.05% polysorbate 20 (PBST) (Sigma) for 1 h at room temperature and then incubated with mouse anti-rMPT83 serum for 2 h at room temperature (1∶500 dilution in PBST). The blots were then washed and incubated with horseradish peroxidase (HRP) conjugated sheep anti-mouse IgG monoclonal antibody for 2 h at room temperature (GE Heathcare, Buckinghamshire, UK). The bands were detected using SuperSignal West Pico Chemiluminescent substrate (Thermo, Waltham, MA) according to the manufacturer’s instructions and the images captured using the Image Station 4000MM imager (Kodak, Rochester, NY) and analysed using the Kodak IM software (Kodak).

### Peptide Preparation

A set of 22 15-mer peptides overlapping by five amino acids spanning the MPT83 protein and the nine peptides for fine mapping (Table 1) were synthesized by Genscript (Piscataway, NJ). The lyophilized peptides were resolubilized according to the supplier’s instructions at 10 mg/ml and stored at −30°C until use.

### Immunization of Animals

Mice were immunized with 100 µg of plasmid DNA resuspended in 100 µl PBS. Fifty microliters were administered by intramuscular injection (i.m.i) into each quadriceps of an anaesthetized mice using an 1 ml insulin syringe with a 27 G needle (Terumo, Somerset, NJ) [48]. The rMPT83 protein was mixed with an adjuvant formulation of dimethyl-dioctadecyl ammonium bromide (DDA) and monophosphoryl lipid A (MPLA) (Sigma) to deliver 10 µg MPT83, 500 µg DDA and 25 µg MPLA in 200 µl and injected subcutaneously into an anaesthetized mice at the base of the tail, twice, two weeks apart with an 1 ml insulin syringe harbouring a 27 G needle [49]. Mice were tested for immune responses four weeks after the final immunization or challenged with *M. tuberculosis* to assess protective efficacy six weeks after the final immunization to minimize any non-specific effects of the DNA plasmid or adjuvant.

### Polyclonal Anti-serum to rMPT83

For the preparation of mouse anti-sera, three mice were immunized with 10 µg of rMPT83 in DDA/MPLA as described above. The mice were bled two weeks after the final immunization, and the specificity of the binding of the sera was assessed by protein immunoblotting.

### Murine T Lymphocyte Responses

Single-cell suspensions were prepared from the spleens of immunized animals and the production of IFN-γ following *in vitro* stimulation was measured. Splenocytes were plated at a density 2×10^5^ cells per well in a 96-well U-bottom tissue culture plate (BD, Franklin Lake, NJ) and incubated in RPMI media (Gibco, Carlsbad, CA) supplemented with 50 µM 2-mercaptoethanol (2-ME) and 10% foetal bovine serum (FBS). The cells were stimulated with rMPT83 protein (10 µg/ml), the overlapping peptides (10 µg/ml), and the controls Concanavalin A (Con A, 3 µg/ml) or media alone. Following incubation at 37°C in 5% CO_2_ for 72 h, the supernatants were collected for analysis.

The IFN-γ sandwich ELISA was performed by coating 96-well ELISA plates (Corning, Lowell, MA) with rat anti-IFN-γ monoclonal antibodies AN18 (1.5 µg/ml, Endogen, Woburn, MA) followed by blocking with 3% BSA. The culture supernatants and the serial dilutions of the IFN-γ standards (Genzyme, Cambridge, MA) ranging from 5000 pg/ml to 39 pg/ml were added to the ELISA plate for 2 h incubation at room temperature. Following washing, the plates were incubated with the secondary antibody XMG1.2-bio (2 µg/ml, Endogen) followed by strepavidin-HRP. The IFN-γ levels were detected by the addition of 2,2′-azino-bis(3-ethylbenzthiazoline-6-sulphonic acid) (ABTS) substrate solution (Sigma) and the absorbance was read at 405 nm and 492 nm with the Multiskan EX plate reader (Thermo) and background absorbance from the unstimulated samples were subtracted from all test samples before analysis.

The numbers of antigen-specific IFN-γ secreting cells were determined by ELIspot. 96-well Immobilon-P plates (Millipore, Bedford, MA) were coated with AN18, 15 µg/ml (Endogen). Single-cell suspensions of splenocytes were plated at a density of 2×10^5^ cells per well and incubated in RPMI media (Gibco) supplemented with 50 µM 2-ME and 10% FBS. The cells were stimulated with rMPT83 protein (10 µg/ml), the overlapping peptides (10 µg/ml), and the controls Con A (3 µg/ml) or media alone. Following 20 h incubation at 37°C in 5% CO_2_, the plates were washed with PBS-Tween20 (0.05% v/v) and incubated for 1 h with XMG1.2-bio (5 µg/ml, Endogen) followed by Avidin-Alkaline Phosphatase (Sigma) conjugate. Spots were visualised by incubation with AP substrate (Biorad). The numbers of spot-forming cells (SPC) were enumerated with an automated ELIspot reader system (AID, Strasburg, Germany). The numbers of SPC from the unstimulated wells were subtracted from all samples before statistical analysis.

### Cell Sorting

Single-cell suspensions were prepared from three spleens per group, pooled and the non-T lymphocytes were depleted by incubation with rat anti-CD19, anti-B220, anti-CD11b and TER-119 monoclonal antibodies and separated using anti-rat IgG microbeads and LS columns by MACS (Miltenyi biotec, Bergisch Gladbach, Germany). The enriched T cells lymphocytes were stained with anti-CD3 PerCP, anti-CD4 Alexafluor 700 and anti-CD8 APC-Cy7 and sorted with FACSaria II (BD). The purities of the sorted CD4^+^ and CD8^+^ T cells was determined by flow cytometry and were 97% and 91%, respectively. In IFN-γ ELIspot assays where purified CD4^+^ or CD8^+^ T cells were used, splenocytes irradiated with 2000 rads of γ radiation were used as syngenic antigen presenting cells at a ratio of 1∶1.

### Human T Lymphocyte Responses

Venous blood was collected with informed consent from patients with active tuberculosis (n = 28) and tuberculin skin test (TST) negative subjects (n = 20) from the Chest Clinic at Royal Prince Alfred Hospital, Sydney. Human peripheral blood mononuclear cells (PBMCs) were purified by Ficoll-Paque gradient centrifugation using Histopaque-1077 (Sigma). The purified PBMCs were cultured at a density of 2.5×10^5^ cells in AIM-V serum free medium (Gibco) supplemented with l-glutamine, gentamicin (10 µg/ml) and streptomycin (50 µg/ml). Cells were stimulated with 10 µg/ml of rMPT83 or *M. tuberculosis* culture filtrate protein (CFP) (provided by NIH contract at Colorado State University, USA), or media alone. After five days incubation at 37°C in 5% CO_2_, the supernatant was collected and the levels of IFN-γ secreted into the supernatant was measured using the OptEIA™ Human IFN-γ ELISA set (BD) in accordance to the manufacturer’s instructions. The cells were then pulsed with 1 µCi of ^3^H-thymidine (MP Biomedicals, Solon, OH) per well for 16 h. The level of incorporated ^3^H-thymidine was determined by liquid scintillation spectroscopy with the Microbeta Luminescence Counter (Perkin-Elmer, Waltham, MA). Specific lymphocyte proliferation was expressed as a delta counts per minute (ΔCPM), calculated as the mean CPM for antigen-stimulated culture wells subtracted by the mean CPM for unstimulated culture wells.

### Statistical Analysis

The significance of the differences in human T cell proliferation and IFN-γ responses were analysed by the Mann-Whitney *U*-test. The differences in murine T cell responses between immunized and non-immunized mice were analysed using an unpaired Student’s *t*-test. The differences in bacterial load were assessed using one-way analysis of variance (ANOVA). Statistical analysis was performed using the GraphPad Prism 5 software (GraphPad Software, La Jolla, CA). The differences were considered significant when the *P* values were ≤0.05.
